# Seroprevalence and risk factors associated with ovine toxoplasmosis in Northeast Brazil

**DOI:** 10.1051/parasite/2013019

**Published:** 2013-05-28

**Authors:** Milena M. Clementino Andrade, Mariangela Carneiro, Andrea D. Medeiros, Valter Andrade Neto, Ricardo W.A. Vitor

**Affiliations:** 1 Departamento de Parasitologia, Instituto de Ciências Biológicas, UFMG, Belo Horizonte MG Brazil; 2 Departamento de Microbiologia e Parasitologia, Centro de Biociências, UFRN Natal RN Brazil

**Keywords:** *Toxoplasma gondii*, sheep, ELISA, seroprevalence

## Abstract

Serum samples of 930 sheep were tested by ELISA to assess the prevalence of anti-*Toxoplasma gondii* antibodies and to identify risk factors associated with the presence of toxoplasmosis in two regions of Rio Grande do Norte (Northeast Brazil), with different climatic conditions. The overall estimated prevalence was 22.1%, with 26.3% and 17.8% positive sheep in Leste Potiguar and Central Potiguar regions, respectively. Among the positive sheep, 18.1% had low-avidity IgG antibodies, suggesting the occurrence of recent toxoplasmosis. The risk factors for toxoplasmosis in sheep were: presence of cats (odds ratio (OR) = 1.55; confidence interval (CI) 95% = 1.11–2.16), age of the animals, with adults presenting a greater chance of infection (OR = 2.44; CI 95% = 1.58–3.75), and the use of running water (OR = 1.61; CI 95% = 1.25–2.09), characterizing the existence of transmission by sporulated oocysts of *T. gondii* in the environment.

## Abstract (fr)

Des échantillons de sérum de 930 moutons ont été analysés par le test ELISA dans le but d’évaluer la prévalence d’anticorps anti-*Toxoplasma gondii* et d’identifier les facteurs de risque associés à la présence de toxoplasmose chez les ovins dans deux régions de l’État de Rio Grande do Norte (Nord-Est du Brésil), ayant des conditions climatiques distinctes. La prévalence estimée était de 22.1 %, dont 26.3 % et 17.8 % de moutons positifs, respectivement, dans les régions Leste Potiguar et Central Potiguar. Chez les moutons positifs, 18,1 % ont présenté des anticorps IgG de basse-avidité, suggérant des événements récents de toxoplasmose. Les facteurs de risque associés à l’infection étaient : la présence de chats (rapport de cotes 1.55 ; intervalle de confiance 95 % 1.11-2.16), l’âge des animaux, les adultes ayant une plus grande chance d’infection (rc 2.44 ; ic 95 % 1.58-3.75), et l’utilisation d’eau courante (rc 1.61; ic 95 % 1.25-2.09), caractérisant ainsi l’existence de transmission par oocystes sporulés de *T. gondii* dans l’environnement.

## Introduction

Toxoplasmosis is a zoonotic disease caused by the intracellular protozoon *Toxoplasma gondii*, capable of infecting homeothermic animals, including sheep and humans [10]. It is transmitted mainly by food or water contaminated by oocysts disseminated by cats and other felids (definitive hosts), raw or undercooked meat containing tissue cysts, or transplacentally [29]. In Natal city (capital of Rio Grande do Norte State), 66% of 190 pregnant women presented IgG antibodies to *T. gondii* [2]. In sheep, *T. gondii* can cause abortions during recent infection, birth defects, and stillbirths [20, 22, 30] resulting in significant economic and reproductive losses, besides public health implications, considering consumption of infected meat, and milk by the population facilitating the zoonotic transmission [22]. In Brazil, seroepidemiological studies on sheep herds in different regions reported an anti-*T. gondii* IgG frequency varying from 7.0% in Paraná to 59% in Fernando de Noronha [9]. Additionally, *T. gondii* IgG avidity can be a good marker of recent infection in sheep [5, 7], without the need for several reagents for the determination of recent toxoplasmosis. Given the global importance of toxoplasmosis in sheep and the few data available on its prevalence in the state of Rio Grande do Norte, limited to only two counties [7, 27], the objective of this study was to determine the seroprevalence of *T. gondii* in sheep from two regions with distinct climatic conditions, as well as to assess the possible risk factors associated with the infection caused by this etiological agent and to detect early toxoplasmosis by research of anti-*T. gondii* IgG avidity.

## Material and methods

### Study area

The state of Rio Grande do Norte, in northeastern Brazil, is situated between the parallels of 4°49′53″ and 6°58′57″ south latitude, and meridians 35°58′03″ and 38°36′12″ west of Greenwich. The annual median temperature of the state is around 25.5 °C, with maximum and minimum of 31.3 °C and 21.1 °C, respectively, and irregular rainfall. The semiarid climate prevails in 60% of the state, characterized by low rainfall, around 400–600 mm per year. The state is divided into four major geographic regions: Agreste Potiguar, Leste Potiguar, Central Potiguar, and Oeste Potiguar. In this study, the animals originate from two regions with distinct climatic characteristics: Leste Potiguar (wet tropical climate) and Central Potiguar (semiarid climate) [14]. In Rio Grande do Norte state, sheep are raised predominantly under a semi-intensive management for meat production.

### Animals and serum

Sample size was determined using the Epi-Info software, version 6.0, based on an assumed prevalence of 29.41% [7] and was calculated using the following parameters: (1) acceptable error range of 0.05; (2) design effect of 2.0 (the samples are not independent, animals were grouped by properties); (3) confidence interval of 95%. The minimum sample size was estimated in 922 sheep (461 per each region).

The survey was conducted from June 2008 to December 2009 and the animals were selected from 25 farms in the state, through a non-probabilistic sampling. No difference was observed between 2 years of sampling. The samples were stratified according to the proportional composition of the herds, defined at least as: four adult females, two adult males, and two lambs (6 months to 1 year old). We did not record if the lambs were twins.

Blood collection was performed in the sheep herd simultaneously with the application of two questionnaires: the first on farm data (region, source of drinking water, food facilities, land use (extensive/intensive), type of flooring, technical monitoring, presence of food trough, type of food trough, presence of water trough, type of water trough, presence of cats) and the second including information on age, sex, and breed of each sheep. Venous puncture of the 930 sheep was performed via a jugular vein and the serum was separated by centrifugation (200 *g* for 5 min) and stored at −20 °C until use.

### ELISA and IgG avidity

ELISA was carried out as described [7]. All the serum samples were tested in duplicate at a dilution of 1:400. Six negative sera and two positive sera, previously tested by IFAT and ELISA [7], were included as control. The cut-off value for each ELISA plate was calculated as the absorbance mean of six serum samples of sheep tested negative for *T. gondii*, plus three standard deviations tested on each plate. Avidity of IgG antibodies was calculated on previously positive samples as the ratio between the mean absorbance for each serum urea-treated (AU) divided by the absorbance mean of the untreated sera (A) expressed in percentage: AU/A × 100 [8]. Avidity values ≥50% indicate chronic toxoplasmosis, while values <50% suggest recent infection [28].

### Statistical analysis

A database was generated using EpiData version 2.1 software and statistical analyses were performed using the Stata Statistical software version 10 SE. Seroprevalence for *T. gondii* and low- and high-avidity IgG antibodies were correlated with age, breed, and regions using the chi-square test. Univariate analysis was used to quantify the association between risk factors and infection by *T. gondii*, using logistic regression. The association measure used was the odds ratio (OR) and confidence interval (CI 95%). Variables that presented *p* < 0.25 in univariate analysis were applied in the multivariate logistic analysis. The *p*-value < 0.05 was considered to build the final model, using the likelihood ratio test to define the model that would best fit the data.

## Results

### Prevalence of toxoplasmosis in sheep

Of the 930 sheep evaluated, 466 (50.1%) were from the Central Potiguar region and 464 (49.9%) from the Leste Potiguar region. Anti-*T. gondii* antibodies were detected in 205 animals (22.1%; CI 95% = 19.5–24.8). We identified 122 (26.3%; CI 95% = 22.4–30.4) and 83 positive sheep (17.8%; CI 95% = 14.5–21.5) in the Leste Potiguar and Central Potiguar regions, respectively (*p* = 0.002).

Table 1 shows seropositivity according to gender, age, and breed. The percentage of 22.1% of positive males (4 lambs and 24 adults) was similar to those observed for the females (25 lambs and 152 adults), 22.0% (*p* = 0.999). Among the lambs, 12.8% were positive for toxoplasmosis, while adults showed seroreactivity of 25.0%. These results show a positive association between the presence of anti-*T. gondii* antibodies and age of sheep (*p* < 0.001). Regarding breed, 22.6% of pure breed, 21.1% of crossbreed, and 19.4% of undetermined breed were positive (*p* = 0.705).

**Table 1.** Variables associated with seroprevalence of *T. gondii* infection in sheep, Rio Grande do Norte, Brazil.

Variables		Examined	Positive (%; CI 95%)	*p*-value
Gender	Males	127	28 (22.1; 15.7–30.0)	0.999
	Females	803	177 (22.0; 19.3–25.0)	
Age	Lambs	226	29 (12.8; 9.1–17.8)	<0.001
	Adults	704	176 (25.0; 21.9–28.3)	
Breed	Crossbreed	730	165 (22.6; 19.7–25.8)	0.705
	Pure	76	16 (21.1; 13.4–31.5)	
	Undetermined	124	24 (19.4; 13.4–27.2)	
Total		930	205 (22.1; 19.5–24.8)	

### IgG avidity

Of the 205 seropositive sheep, 168 (81.9%) had high-avidity IgG antibodies and 37 (18.1%), low-avidity antibodies. No statistically significant difference (*p* > 0.05) was found between the frequency of low-avidity antibodies and breed or region. The avidity rates available by age showed significant differences (*p* < 0.05). The frequency of lambs presenting low-avidity antibodies (37.9%) was higher than that of adult animals (14.8%) (*p* = 0.004).

### Risk factors

Variables associated with *T. gondii* infection in univariate analysis were: source of drinking water (running water or still water); type of flooring (dirt or cemented floor); location of drinking trough (animals drink inside the facilities or directly from water source); type of water trough (cement or plastic); type of food trough (cement or wood); presence of cats; food-storage facility, and age. The variables remaining in the final model were: presence of cats, sheep age (adult animals), and use of unexposed water obtained from deep well (Table 2). The presence of cats on the farms increased the chances of sheep to become infected with *T. gondii* (OR = 1.55, CI 95% = 1.11–2.16). Adult sheep had a higher risk of infection when compared to lambs (OR = 2.44; CI 95% = 1.58–3.75). Sheep from farms where the water offered was obtained from running water systems sources had a higher risk to become infected with *T. gondii* than those from farms with still-water sources (OR = 1.61; CI 95% = 1.25–2.09).

**Table 2.** Risk factors associated with toxoplasmosis in sheep, Rio Grande do Norte, Brazil.

Risk factors	OR	CI 95%	*p*
Water source			
Still water	1	–	–
Running water	1.61	1.25–2.09	0.000
Presence of cats			
No	1	–	–
Yes	1.55	1.11–2.16	0.009
Age			
Lambs	1	–	–
Adults	2.44	1.58–3.75	0.000

## Discussion

Seroprevalence of anti-*T. gondii* antibodies observed in sheep from the state of Rio Grande do Norte was 22.1%. This value is close to those previously observed in the same state, 29.4% and 20.7% of the sheep tested positive in the municipalities of Lajes and Mossoro, respectively [7, 27]. This value is also similar to those found in Italy – 28.5% [11], Finland – 24.6% [15], Netherlands – 27.8% [21], and China – 29.8% [17]. However, it was higher than that found in Nigeria (6.7%) [16]. In contrast, our data were lower than those observed in the Czech Republic – 59% [3] and in India – 44.1% [6]. The differences observed between these rates of prevalence may be related to the serological methods used in these studies (it should be taken into account that the different tests used to obtain these data are not standardized), system of exploitation, presence of definitive hosts, and climatic variables.

Among the regions studied in Rio Grande do Norte, seroprevalence varied from 17.8% in the Central Potiguar region to 26.3% in the Leste Potiguar region. These results show that climatic differences may influence on the spread of toxoplasmosis in sheep, considering that the Leste Potiguar region presents more favorable climatic conditions to the development and maintenance of *T. gondii* oocysts in the environment. Oocysts can survive in the environment for months, depending on moisture and temperature [30]. Thus, low humidity and high temperatures, typical of the Central Potiguar region, are deleterious to oocysts. This fact was also demonstrated in the state of Bahia, Brazil [24], where a prevalence of 12.5% was observed in the “caatinga” arid region and 26.92% in the humid area bordering the Atlantic Coast. This fact suggests that climate characteristics of dry regions are likely to decrease the chance of oocyst survival, generally resulting in a low prevalence of toxoplasmosis.

Our results show a positive association between age and seroprevalence. Adults animals showed higher seroreactivity than lambs. This is likely due to increasing opportunities of exposure to the infectious agent [12, 13]. No relationship was found between sheep gender and seropositivity for toxoplasmosis, confirming previous studies [4, 5, 27]. No significant difference was found among breed categories analyzed. These data corroborate previous results [25, 31]. However, in the state of Pernambuco, Brazil, it was observed higher prevalence in crossbred sheep [26]. Probably, this fact is due to the poor hygienic-sanitary management adopted in the farms where crossbred sheep are raised. Our results show that less than 20% of the seropositive sheep have low-avidity anti-*T. gondii* IgG antibodies, suggesting recently acquired toxoplasmosis. Lambs have low-avidity antibodies with greater frequency.

The risk factors identified for sheep toxoplasmosis in the regions studied in the state of Rio Grande do Norte were: age, presence of cats, and use of running water. Adult sheep are 2.44 times more likely (CI 95% = 1.58–3.75) to be infected, compared to the younger animals. As noted earlier, older animals present a higher prevalence due to the increased contact time with oocysts of *T. gondii* in the environment [16, 23]. The presence of cats on the farms increased 1.55 (CI 95% = 1.11–2.16) the risk for sheep to become infected with *T. gondii*. This association is likely due to a greater environmental contamination with oocysts eliminated in the feces of these felids [1, 18]. Sheep reared on farms where drinking water was obtained from a running source (continuous replacement of water, obtained from a subterraneous source) had a 1.61 greater risk (CI 95% = 1.25–2.09) to acquire toxoplasmosis than the animals from properties with still-water sources (partially stagnant water, without continuous replacement). This finding is corroborated by Pinheiro et al. [23] who observed that running water is a risk factor for toxoplasmosis. However, our results are discordant from those observed in southeastern Brazil [19]. These authors demonstrated that sheep from flocks using lake water are 1.67 times more likely to be infected compared to animal that drink spring water. Our results are different from expected, but we do not know the reasons. Due to a higher chance of exposure to cat feces, still water would be more likely to be contaminated with oocysts of *T. gondii* than running water. Anyway, drinking water must also be considered as an important means of agent transmission, acting as a disseminator of oocysts in flocks of sheep.

Based on the results of this study, it was concluded that *T. gondii* infection is common in Brazilian sheep from the state of Rio Grande do Norte. Age of the animals, presence of cats, and the use of unexposed source of water are risk factors for toxoplasmosis in sheep.
